# Matching‐adjusted indirect comparison of isatuximab plus carfilzomib and dexamethasone with daratumumab plus lenalidomide and dexamethasone in relapsed multiple myeloma

**DOI:** 10.1002/cam4.5584

**Published:** 2023-02-01

**Authors:** Joshua Richter, Peggy L. Lin, Viviana Garcia‐Horton, Patricia Guyot, Erin Singh, Zheng‐Yi Zhou, Mark Sievert, Riley Taiji

**Affiliations:** ^1^ Icahn School of Medicine at Mount Sinai New York New York USA; ^2^ Sanofi Cambridge Massachusetts USA; ^3^ Analysis Group New York New York USA; ^4^ Sanofi Chilly‐Mazarin France; ^5^ Analysis Group London UK

**Keywords:** comparative efficacy, daratumumab, isatuximab, matching‐adjusted indirect comparison, relapsed refractory multiple myeloma

## Abstract

**Backgound:**

Lenalidomide‐based regimens are commonly used for early relapse in patients with relapsed and/or refractory multiple myeloma (RRMM) receiving at least one prior line of therapy. In the absence of head‐to‐head comparison, matching‐adjusted indirect comparison (MAIC) was conducted to demonstrate efficacy and safety of isatuximab+carfilzomib+dexamethasone (Isa‐Kd) versus daratumumab + lenalidomide + dexamethasone (Dara‐Rd) in RRMM.

**Methods:**

Patient‐level data from IKEMA trial (Isa‐Kd, *n* = 179) were matched to aggregate data from POLLUX (Dara‐Rd, *n* = 286). Hazard ratios (HR) and 95% confidence intervals (CI) for progression‐free survival (PFS) and overall survival (OS) were generated by weighted Cox proportional hazard models. Odds ratios (OR), 95% CI, and *p*‐value were calculated for ≥very good partial response (≥VGPR) and treatment‐emergent adverse events (TEAEs).

**Results:**

After matching, no significant differences were observed between Isa‐Kd and Dara‐Rd in baseline characteristics except for patients with >3 prior lines (0.0% vs. 4.9%). Isa‐Kd showed significantly better PFS (HR [95% CI]: 0.46 [0.24–0.86]; *p* = 0.0155), statistically non‐significant improvement favoring Isa‐Kd in OS (0.47 [0.20–1.09]; 0.0798), and ≥VGPR (OR [95% CI]: 1.53 [0.89–2.64]; *p* = 0.1252) than Dara‐Rd. Odds of occurrence were significantly lower for some all‐grade and grade 3/4 TEAEs with Isa‐Kd than Dara‐Rd.

**Conclusion:**

These results support Isa‐Kd as an efficacious treatment for early relapse in non‐lenalidomide refractory patients.

## Graphical abstract

Matching‐adjusted indirect comparison results for Isa‐Kd vs. Dara‐Rd: Kaplan‐Meier curves of progression‐free survival before matching and after inclusion criteria selection (A) and after matching (B); Kaplan‐Meier curves of overall survival before matching and after inclusion criteria selection (C) and after matching (D). CI, confidence interval; Dara‐Rd, daratumumab + lenalidomide + dexamethasone; HR, hazard ratio; Isa‐Kd, isatuximab + carfilzomib + dexamethasone. *Statistically significant at 0.05 level.

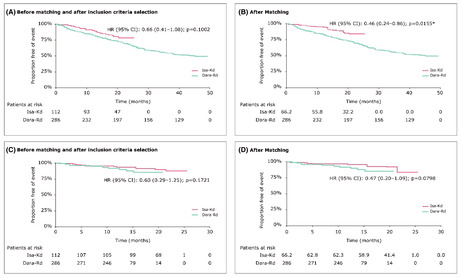

## 1 INTRODUCTION

Despite being a rare form of cancer, multiple myeloma (MM) is the second most common hematologic malignancy characterized by excessive production of malignant monoclonal plasma cells.^1^, ^2^, ^3^ In 2020, nearly 176,400 newly diagnosed cases and about 117,000 MM‐related deaths were reported globally.^4^ The US surveillance, Epidemiology, and End Results data registry estimated 34,920 new cases with 12,410 deaths related to MM in 2021.^5^ The incidence rate of MM in the United Kingdom is projected to increase by 11% between 2014 and 2035 to 12 cases per 100,000 by 2035.^6^, ^7^

The treatment landscape of MM has evolved over the last 10 years with the incorporation of immunomodulatory imid drugs (IMiDs, such as lenalidomide pomalidomide) and proteasome inhibitors (such as bortezomib and carfilzomib), which has resulted in improved overall survival (OS) and a prognosis with nearly 55.6% of diagnosed patients surviving for ≥5 years.^5^, ^8^, ^9^ The approval of anti‐CD38 monoclonal antibodies (mAB) has further improved clinical outcomes in patients with relapsed and/or refractory multiple myeloma (RRMM).^10^ The CD38 type II transmembrane glycoprotein is abundantly expressed on MM cells, thus providing a unique target for CD38 antibodies in patients with MM.^11^ Currently, isatuximab (anti‐CD38 IgG1 mAB) and daratumumab (anti‐CD38 human IgG‐κ mAB) are the only two anti‐CD38 mABs approved for the treatment of RRMM. Based on the results of the phase 3 IKEMA trial (NCT03275285), isatuximab in combination with carfilzomib and dexamethasone (Isa‐Kd) was approved by the US FDA on March 31, 2021, for the treatment of adult patients with RRMM who have received one to three prior lines of therapy (LoT),^12^, ^13^, ^14^ and by the European Medicine Agency on April 19, 2021, for the treatment of adult patients with MM who have received at least one prior therapy.^15^ Daratumumab, in combination with lenalidomide and dexamethasone (Dara‐Rd), was approved by the US FDA on November 21, 2016 and by European Medicine Agency on May 20, 2016, for the treatment of adult patients with RRMM^16^, ^17^, ^18^ and MM^19^ who have received at least one prior therapy, respectively. More recently, daratumumab, in combination with carfilzomib and dexamethasone (Dara‐Kd), was approved by the US FDA on August 20, 2020 to treat adult patients with RRMM who have received one to three LoT.^20^

The choice of a treatment regimen for patients with RRMM depends upon several factors, such as response toward prior therapies, patient condition, expected effectiveness, and tolerability.^21^ The current literature highlights that lenalidomide‐based regimens are frequently used in non‐lenalidomide‐refractory early relapse patients until progression in the second line (2L+) setting.^22^, ^23^, ^24^ A Flatiron Health Database study revealed that lenalidomide‐based regimens are the most commonly used treatment option in patients with MM, as monotherapy or combination therapy (with PI or anti‐CD38 mAB), despite experiencing relapse on lenalidomide.^22^ For optimum effect, doublet or triplet‐based regimens are usually preferred over single agents for patients with RRMM.^21^ In the 2L+ setting, triple combination therapy with mAB + IMiD + dexamethasone, such as Dara‐Rd is a go‐to regimen for patients at first relapse, and it is frequently used in non‐lenalidomide‐refractory patients until progression, whereas Isa‐Kd is usually considered for lenalidomide‐refractory patients.^23^, ^24^, ^25^

Despite advances in therapy with improved survival outcomes, MM remains largely incurable, with patients continuously relapsing over the course of the disease.^9^, ^26^, ^27^ Over time, patients can become refractory or resistant to the existing therapies, that is, develop RRMM.^9^, ^26^, ^27^ A retrospective evaluation of data on patients with MM treated at Mayo Clinic between January 2006 and December 2014 revealed that 82 (16.0%) patients relapsed within 12 months (median: 8.0 (95% CI: 6.3–8.9)) of starting initial therapy with autologous stem cell transplant and induction regimens, such as IMiDs, proteasome inhibitors, or both.^28^ An International Myeloma Working Group study that enrolled 543 patients with MM who had received at least three prior LoT (median: 4 [3–13]) highlighted that the median survival for the entire cohort was relatively low, that is, 13 months.^29^ Likewise, a real‐world retrospective study of patients with MM who were refractory to IMiDs and proteasome inhibitors or who had received three or more prior LoT, including a PI and an IMiD, reported a median OS of only 8 months approximately.^30^

Evidence of comparative efficacy is crucial to demonstrate treatment value and aid clinical decision‐making in choosing the optimal treatment regimen. In the absence of published evidence from head‐to‐head clinical trials and without a common comparator between the clinical trials, matching‐adjusted indirect comparison (MAIC) was used to compare the efficacy and safety of two approved anti‐CD38 mABs, that is, Isa‐Kd versus Dara‐Rd, to inform treatment choice in patients with RRMM at their first relapse. The National Institute for Health and Care Excellence support document outlines the MAIC approach for comparing relative treatment effects between two interventions^31^ when direct treatment comparison is not feasible.^32^, ^33^ Recently, the MAIC methodology has provided valuable insights into the relative efficacy of different oncology treatment options, including RRMM.^34^, ^35^

The primary objective of this MAIC analysis was to compare the progression‐free survival (PFS), OS, and very good partial response (VGPR) or better of Isa‐Kd with that of Dara‐Rd. The secondary objective was to assess the safety of Isa‐Kd versus that of Dara‐Rd in patients with RRMM.

## 2 METHODS

### 2.1 Data sources

In this MAIC analysis, individual patient‐level data (IPD) for Isa‐Kd were obtained from the IKEMA trial (median follow‐up: 20.7 months).^14^, ^36^ In brief, IKEMA is a multinational, prospective, randomized, open‐label, parallel‐group, phase 3 study conducted at 69 study centers in 16 countries across North America, South America, Europe, and the Asia‐Pacific region. Patients with RRMM, aged at least 18 years, who had received one to three previous LoT and had measurable evidence of disease (serum M‐protein ≥0.5 g/dL or urine M‐protein ≥200 mg/24 h) were eligible for enrollment in the IKEMA trial. The complete list of inclusion and exclusion criteria of the IKEMA trial has been published previously.^14^, ^36^ A total of 302 eligible patients were randomly assigned in a 3:2 ratio to receive either Isa‐Kd (i.e., isatuximab group; *n* = 179) or carfilzomib and dexamethasone (Kd) (i.e., control group; *n* = 123). For patients assigned to the isatuximab group, isatuximab at a dose of 10 mg/kg was administered intravenously (i.v.) on days 1, 8, 15, and 22 in the first 28‐day cycle and on days 1 and 15 in the following cycles. Patients in both treatment groups received carfilzomib (20 mg/m^2^, i.v.) on days 1 and 2 of cycle 1; 56 mg/m^2^ on days 8, 9, 15, and 16 of cycle 1; and then 56 mg/m^2^ on days 1, 2, 8, 9, 15, and 16 of the following cycles. Dexamethasone (20 mg, i.v. or orally) was administered on days 1, 2, 8, 9, 15, 16, 22, and 23 of every cycle.^14^, ^36^ The primary efficacy endpoint of IKEMA was PFS, and key secondary efficacy endpoints were overall response rate, rate of VGPR or better, and OS. The other secondary endpoints, including safety, were also assessed.^14^, ^36^

For Dara‐Rd, the published aggregate data were obtained from the POLLUX trial publications.^18^, ^37^ In brief, POLLUX is a randomized, open‐label, multicenter, active‐controlled, parallel‐group, phase 3 study conducted at 135 sites in 18 countries across North America, Europe, and the Asia‐Pacific region. Patients with RRMM who had received one or more lines of previous therapy and had measurable disease at screening were eligible for enrollment in the POLLUX trial. The complete list of inclusion and exclusion criteria of the POLLUX trial has been published previously.^18^ A total of 569 eligible patients were randomly assigned in a 1:1 ratio to receive either Dara‐Rd (i.e., daratumumab group; *n* = 286) or lenalidomide and dexamethasone (Rd) (i.e., control group; *n* = 283). For patients who were assigned to the daratumumab group, daratumumab at a dose of 16 mg/kg was administered i.v. on days 1, 8, 15, and 22 for 8 weeks during cycles 1 and 2; every 2 weeks (on days 1 and 15) for 16 weeks (cycles 3 through 6); and every 4 weeks subsequently. Patients in both treatment groups received lenalidomide (25 mg, orally) on days 1 to 21 of each cycle and dexamethasone (40 mg, orally) once every week.^18^ The primary efficacy endpoint of POLLUX was PFS, and secondary efficacy endpoints included time to progression, overall response rate, rate of VGPR or better, rate of complete response (CR) or better, and OS. Additionally, adverse events were also monitored.^18^

### 2.2 Feasibility assessment

A feasibility assessment was conducted to evaluate key points of similarity and heterogeneity (i.e., cross‐trial imbalances) between the IKEMA and POLLUX trials, including trial design, treatment arms, patient population, inclusion and exclusion criteria, sample size, outcome definitions, and assessments. This assessment revealed that the study design, outcome definitions, and eligibility criteria were broadly similar between the IKEMA and POLLUX trials. Furthermore, there was sufficient overlap between the summarized baseline characteristics reported in the primary POLLUX trial publication^18^ and the available measures in IKEMA IPD.^37^, ^38^ Therefore, an unanchored MAIC between Isa‐Kd and Dara‐Rd was deemed feasible based on the IKEMA and corresponding POLLUX trial/arm data for PFS, OS, VGPR or better, and treatment‐emergent adverse events (TEAEs). However, some key differences were identified in the eligibility criteria between these two trials. Therefore, to emulate the POLLUX trial population, additional eligibility criteria of POLLUX (creatinine clearance ≥30 mL/min, hemoglobin >7.5 g/dL, platelet count ≥75 × 10^9^/L, and non‐lenalidomide‐refractory) were applied to the IKEMA Isa‐Kd IPD.

### 2.3 Comparison of baseline characteristics and outcomes before matching

The baseline characteristics were summarized and compared between the Isa‐Kd population of IKEMA and the Dara‐Rd population of POLLUX, using Wald and chi‐square tests (or Fisher's exact test for small frequencies) for continuous and categorical variables, respectively.

The outcomes of interest assessed in the MAIC analysis were based on the primary and secondary endpoints of the IKEMA and POLLUX trials. The endpoints included PFS, OS, VGPR or better, as well as all‐grade and grade 3/4 TEAEs. For PFS^39^ and OS,^18^ IPD for Isa‐Kd was used from the IKEMA trial, and data from the published Kaplan‐Meier (KM) curves for Dara‐Rd were extracted via digitization software.^40^ Based on the extracted survival curves, reported numbers of events, and patients at risk at various time points in the publications, a pseudo‐IPD was reconstructed using the approach developed by Guyot et al.^41^ Before matching, PFS and OS were summarized using the KM curves in addition to the median PFS and OS (in months), and log‐rank tests were conducted to compare the Isa‐Kd and Dara‐Rd arms. The HR and corresponding 95% CI and *p*‐values were estimated from Cox proportional hazards models.

The VGPR or better (calculated as the proportion of patients with CR, stringent CR, or VGPR as the best overall response) was obtained from the IPD for Isa‐Kd and from the POLLUX publication with the closest follow‐up to the IKEMA trial that reported this outcome.^14^, ^37^ ORs were reported, and the corresponding 95% CI and *p*‐value were calculated using the Wald tests to compare between comparators before matching.

The all‐grade TEAEs and all‐grade 3 or 4 TEAEs with rates >5% reported for the Dara‐Rd arm of the POLLUX trial^37^ were included. The data collection and analysis of four hematological adverse events (i.e., neutropenia, anemia, thrombocytopenia, and lymphopenia) were different in POLLUX and IKEMA. In POLLUX laboratory, data were reported as TEAEs while in IKEMA all laboratory tests (normal and abnormal) were to be reported and the worst grade was calculated from all collected laboratory values. Therefore, they were excluded from the MAIC analyses. The OR, 95% CI, and *p*‐values were calculated before matching.

### 2.4 Matching average baseline characteristics

The aggregate baseline characteristics (average or proportions) were matched between the Isa‐Kd and Dara‐Rd patients. Specifically, individual patients in the IKEMA trial were assigned weights such that the weighted mean (or proportion) baseline characteristics for patients in the Isa‐Kd arm exactly matched those reported for patients in the Dara‐Rd arm, and each patient's weight was equal to their estimated odds (propensity) of being in the POLLUX trial versus the IKEMA trial. The weights meeting these conditions were obtained from a logistic regression model for the propensity of enrollment in the POLLUX trial versus the IKEMA trial. All matched‐on baseline characteristics were included as predictors in the model.

The matched‐on baseline characteristics were prioritized for inclusion in the Isa‐Kd versus Dara‐Rd MAIC by internal medical and statistics experts based on clinical importance (i.e., the potential for treatment modification) and major trial differences. The matched‐on characteristics included age (≤64, 65–74, and ≥75 years), the Eastern Cooperative Oncology Group performance status (0; 1–2), number of prior therapy lines (1 or ≥2), disease stage (I, II, or III per the International Staging System) at entry, cytogenetic risk (standard, high, or unknown), prior treatment (PI, lenalidomide, and IMiD), and refractory status (PI‐refractory only or IMiD‐refractory only). The other baseline characteristics reported/included but not matched were gender (%male), race/ethnicity, geographic region, creatinine clearance levels, previous treatments (autologous stem cell transplant, or alkylating agent), refractoriness to PI, IMiD, refractoriness to the previous line, and years from diagnosis to randomization.

### 2.5 Comparisons of baseline characteristics and outcomes after matching

Post‐matching adjustments, the baseline characteristics were compared between the Isa‐Kd and Dara‐Rd arms; *p*‐values for continuous and categorical variables were calculated using the Wald tests. Effective sample size (ESS) was also calculated.

For PFS and OS, the KM curves were estimated for Isa‐Kd after matching by incorporating the matching weights and compared with Dara‐Rd. The median PFS or OS was calculated from the estimated KM curves. Weighted log‐rank tests were conducted to compare Isa‐Kd with Dara‐Rd after matching. The HRs, including 95% CI and *p*‐values for PFS and OS, were estimated using a weighted Cox proportional hazards model based on the weighted sample of Isa‐Kd and the pseudo‐IPD of the comparator. The proportional hazards assumption tests were performed based on the scaled Schoenfeld residuals, and the corresponding *p*‐values were reported. The OR and corresponding 95% CI and *p*‐values for VGPR or better were calculated using the Wald tests. Likewise, the OR, 95% CI, and *p*‐values were calculated for all‐grade and grade 3/4 TEAEs.

## 3 RESULTS

### 3.1 Patient demographic and baseline characteristics

The original sample size of the Isa‐Kd arm of the IKEMA trial was 179, of which 112 patients were included in this MAIC analysis and were considered for weighting following the imposition of additional eligibility criteria of POLLUX. A total of 286 patients were included in the Dara‐Rd arm of the POLLUX trial (Table 1).

**TABLE 1:** Baseline patient characteristics before and after matching in matching‐adjusted indirect comparison results of Isa‐Kd versus Dara‐Rd^a^.

Characteristics	Before matching			After matching		
	Isa‐Kd	Dara‐Rd	*p*‐value*	Isa‐Kd	Dara‐Rd	*p*‐value**
	*N* = 112	*N* = 286		ESS = 66.2	*N* = 286	
Age (years)						
Mean ± SD	64.2 ± 9.1	64.4 ± 9.0	0.8550	64.3 ± 9.1	64.4 ± 9.0	0.8968
<65^b^, *n* (%)	50 (44.6%)	133 (46.5%)	0.8235	30.8 (46.5%)	133 (46.5%)	~
65–74^b^, *n* (%)	51 (45.5%)	124 (43.4%)	0.7783	28.7 (43.4%)	124 (43.4%)	~
≥75^b^, *n* (%)	11 (9.8%)	29 (10.1%)	1.0000	6.7 (10.1%)	29 (10.1%)	~
Gender						
Male, *n* (%)	59 (52.7%)	173 (60.5%)	0.1908	37.7 (57.0%)	173 (60.5%)	0.6083
Race/ethnicity, n (%)						
White	82 (73.2%)	207 (72.4%)	0.9654	50.7 (76.6%)	207 (72.4%)	0.4640
Asian	18 (16.1%)	54 (18.9%)	0.61	9.7 (14.6%)	54 (18.9%)	0.3761
Black	3 (2.7%)	5 (1.7%)	0.6919	2.9 (4.4%)	5 (1.7%)	0.3689
Other	9 (8.0%)	20 (7.0%)	0.8843	2.9 (4.4%)	20 (7.0%)	0.2715
Geographic region, *n* (%)						
Europe	56 (50.0%)	150 (52.5%)	0.743	35.7 (53.9%)	150 (52.5%)	0.8283
North America	14 (12.5%)	38 (13.3%)	0.9649	10.9 (16.5%)	38 (13.3%)	0.5568
Rest of the world	42 (37.5%)	98 (34.3%)	0.6235	19.5 (29.5%)	98 (34.3%)	0.4363
ECOG performance status, *n* (%)						
0^b^	61 (54.5%)	139 (48.6%)	0.3470	32.2 (48.6%)	139 (48.6%)	~
1 or 2^b^	50 (44.6%)	147 (51.4%)	0.2710	34.0 (51.4%)	147 (51.4%)	~
Number of previous lines of therapy, n (%)						
1^b^	66 (58.9%)	149 (52.1%)	0.2637	34.5 (52.1%)	149 (52.1%)	~
2	34 (30.4%)	85 (29.7%)	0.9976	20.3 (30.7%)	85 (29.7%)	0.8804
3	12 (10.7%)	38 (13.3%)	0.5974	11.4 (17.2%)	38 (13.3%)	0.4899
>3	0 (0.0%)	14 (4.9%)	0.0134****	0.0 (0.0%)	14 (4.9%)	0.0134****
2 or 3	46 (41.1%)	123 (43.0%)	0.8115	31.7 (47.9%)	123 (43.0%)	0.4759
≥2^b^	46 (41.1%)	137 (47.9%)	0.2637	31.7 (47.9%)	137 (47.9%)	~
≥3	12 (10.7%)	52 (18.2%)	0.0945	11.4 (17.2%)	52 (18.2%)	0.8685
Disease stage at study entry, International staging system (ISS), *n* (%)						
I	61 (54.5%)	137 (47.9%)	0.2864	32.0 (48.3%)	137 (47.9%)	0.9538
II	37 (33.0%)	93 (32.5%)	1.0000	21.3 (32.1%)	93 (32.5%)	0.9503
III^b^	14 (12.5%)	56 (19.6%)	0.1280	13.0 (19.6%)	56 (19.6%)	~
I or II^b^	98 (87.5%)	230 (80.4%)	0.1280	53.2 (80.4%)	230 (80.4%)	~
Cytogenetic risk, *n* (%)						
High^b^ ^,^ ***	27 (24.1%)	35 (12.2%)	0.0054****	8.1 (12.2%)	35 (12.2%)	~
Standard^b^	72 (64.3%)	193 (67.5%)	0.6242	44.7 (67.5%)	193 (67.5%)	~
Unknown^b^	13 (11.6%)	58 (20.3%)	0.0592	13.4 (20.3%)	58 (20.3%)	~
Creatinine levels, *n* (%)						
≤60 mL/min	29 (25.9%)	80 (28.0%)	0.7693	18.3 (27.7%)	80 (28.0%)	0.9683
>60 mL/min	83 (74.1%)	199 (69.6%)	0.4407	47.9 (72.3%)	199 (69.6%)	0.6715
Missing	0 (0.0%)	7 (2.4%)	0.1982	0.0 (0.0%)	7 (2.4%)	0.1982
Previous treatments, *n* (%)						
ASCT	69 (61.6%)	180 (62.9%)	0.8955	36.4 (54.9%)	180 (62.9%)	0.2351
Alkylating agents	105 (93.8%)	268 (93.7%)	1.0000	61.2 (92.4%)	268 (93.7%)	0.7307
PI^b^	104 (92.9%)	245 (85.7%)	0.0728	56.8 (85.7%)	245 (85.7%)	~
IMiD^b^	70 (62.5%)	158 (55.2%)	0.2289	36.6 (55.2%)	158 (55.2%)	~
Lenalidomide^b^	12 (10.7%)	50 (17.5%)	0.1283	11.6 (17.5%)	50 (17.5%)	~
Refractory disease, *n* (%)						
Refractory to PI only^b^	19 (17.0%)	57 (19.9%)	0.5926	13.2 (19.9%)	57 (19.9%)	~
Refractory to IMiD only^b^	11 (9.8%)	10 (3.5%)	0.0221****	2.3 (3.5%)	10 (3.5%)	~
Refractory to PI and IMiD	7 (6.3%)	7 (2.4%)	0.1213	4.2 (6.3%)	7 (2.4%)	0.1617
Refractory to previous line	39 (34.8%)	80 (28.0%)	0.2223	25.8 (39.0%)	80 (28.0%)	0.0968
Time since diagnosis						
Years from diagnosis to randomization (≥Dara‐Rd median of 3.5)	50 (44.6%)	143 (50.0%)	0.3953	33.5 (50.6%)	143 (50.0%)	0.9356

Abbreviations: ASCT, autologous stem cell transplant; Dara‐Rd, daratumumab + lenalidomide + dexamethasone; ECOG, Eastern Cooperative Oncology Group; IMiD, immunomodulatory drug; ESS, effective sample size; Isa‐Kd, isatuximab + carfilzomib + dexamethasone; MAIC, matching‐adjusted indirect comparison; min, minute; PI, proteasome inhibitor; SD, standard deviation.

^a^ Means and standard deviations are shown for continuous characteristics; percentages are shown for categorical characteristics.

^b^ Characteristics matched on in MAIC.

* Before matching, *p*‐values for continuous variables are calculated using the Wald test. *p*‐Values for categorical variables are calculated using the chi‐square test. The Fisher's exact test is used for categorical variables with small frequency (i.e., *n* < 5)

** After matching: All *p*‐values are calculated using the Wald test. The *p*‐value is not calculated for matched variables; instead, a “~” is shown

*** High‐risk cytogenetic group in both POLLUX and IKEMA includes patients with del(17p) and/or translocation t(4;14) and/or translocation *t*(14;16)

**** *p*‐Value < 0.05.

Before matching but after applying the POLLUX inclusion criteria, statistically significant differences (*p* < 0.05) were observed between the Isa‐Kd and Dara‐Rd populations in the percentage of patients with more than three prior LoT (0.0% vs. 4.9%), high cytogenetic risk (24.1% vs. 12.2%), and who were refractory to IMiD only (9.8% vs. 3.5%). After MAIC weighting, all baseline characteristics were well balanced between Isa‐Kd and Dara‐Rd populations, except for patients with more than three prior LoT (0.0% vs. 4.9%). Additionally, a balance was also maintained in the factors, which were not matched‐on as indicated by the *p*‐values.

Based on the weighting of the observations, the ESS adjusts the sample size to show the amount of overlap in patient baseline characteristics between the study populations included in the MAIC analysis.^42^ After matching, the sample size was reduced by 40.8%. The ESS of Isa‐Kd was 66.2, reflecting the approximate number of patients with an overlap of baseline characteristics with theDara‐Rd arm of the POLLUX trial (*n* = 286).

### 3.2 Progression‐free survival

**FIGURE 1:**
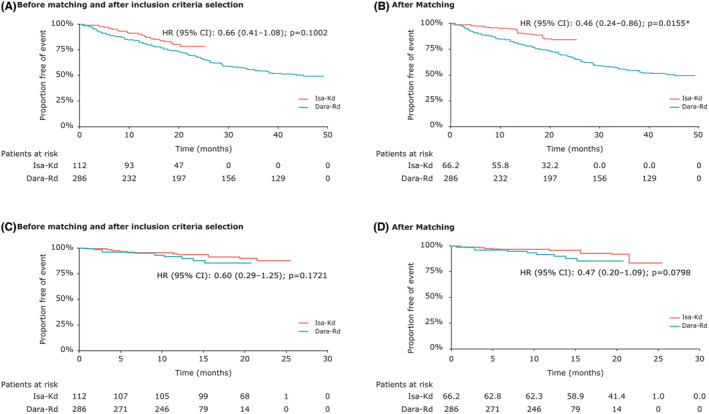
Matching‐adjusted indirect comparison results for Isa‐Kd versus Dara‐Rd: Kaplan‐Meier curves of progression‐free survival before matching and after inclusion criteria selection (A) and after matching (B); Kaplan‐Meier curves of overall survival before matching and after inclusion criteria selection (C) and after matching (D). CI, confidence interval; Dara‐Rd, daratumumab + lenalidomide + dexamethasone; HR, hazard ratio; Isa‐Kd, isatuximab + carfilzomib + dexamethasone. *Statistically significant at 0.05 level.

The proportional hazards assumption was tested before (*χ* ^2^ = 0.01, *p* = 0.92) and after (*χ* ^2^ = 1.47, *p* = 0.23) matching, and the results showed that the assumption was not violated (Figure 1).

The pre‐ and post‐matched PFS HRs of Isa‐Kd versus Dara‐Rd were 0.66 (95% CI: 0.41–1.08; *p* = 0.1002, Figure 1A) and 0.46 (95% CI: 0.24–0.86; *p* = 0.0155, Figure 1B). A significant difference was observed in the PFS HR between the two treatments after matching in favor of Isa‐Kd.

### 3.3 Overall survival

The proportional hazards assumption was tested before (*χ* ^2^ = 0.11, *p* = 0.74) and after (*χ* ^2^ = 0.19, *p* = 0.66) matching, and the results showed that the assumption was not violated (Figure 1).

The pre‐ and post‐matched OS HRs of Isa‐Kd versus Dara‐Rd were 0.60 (95% CI: 0.29–1.25; *p* = 0.1721, Figure 1C) and 0.47 (95% CI: 0.20–1.09; *p* = 0.0798, Figure 1D). No significant differences were observed in the OS HR between the two treatments before and after matching. However, a numerical trend was observed for OS in favor of Isa‐Kd compared with Dara‐Rd after matching adjustment.

### 3.4 Very good partial response or better

Before matching adjustment, a similar proportion of patients receiving Isa‐Kd and Dara‐Rd achieved VGPR or better (78.6% vs. 78.7%; OR = 1.00; *p* = 0.9837). Post‐MAIC weighting, a statistically non‐significant improvement in the rate of VGPR or better was observed in favor of Isa‐Kd (84.9% vs. 78.7%; OR = 1.53, *p* = 0.1252) compared with Dara‐Rd (Table 2).

**TABLE 2:** Matching‐adjusted indirect comparison results: VGPR or better of Isa‐Kd and Dara‐Rd before and after matching.

Arm	Before matching			After matching		
	VGPR or better	95% CI		VGPR or better	95% CI	
Dara‐Rd	78.7%	(73.4%–83.3%)		78.7%	(73.4%–83.3%)	
Isa‐Kd	78.6%	(70.4%–85.5%)		84.9%	(76.1%–90.9%)	

	Before matching			After matching		
	Odds ratio	95% CI	*p*‐value	Odds ratio	95% CI	*p*‐value
Isa‐Kd versus Dara‐Rd	1.00	(0.64–1.54)	0.9837	1.53	(0.89–2.64)	0.1252

Abbreviations: CI, confidence interval; Dara‐Rd, daratumumab + lenalidomide + dexamethasone; Isa‐Kd, isatuximab + carfilzomib + dexamethasone; VGPR, very good partial response.

### 3.5 Safety outcomes

After MAIC weighting, TEAEs leading to treatment discontinuations were higher among patients receiving Dara‐Rd than those receiving Isa‐Kd (12.0% vs. 9.0%), although the difference was not significant (*p* = 0.4712) (Table 3). Treatment with Isa‐Kd, after matching, was associated with significantly (*p* < 0.05) lower rates of all‐grade constipation, cough, diarrhea, fatigue, febrile neutropenia, muscle spasms, nasopharyngitis, nausea, and pyrexia than Dara‐Rd (Table 3). Conversely, a higher rate of asthenia, bronchitis, dyspnea, headache, pneumonia, and upper respiratory tract infections was observed in the Isa‐Kd arm than in the Dara‐Rd arm, although the difference was not significant. Likewise, the proportion of patients reporting back pain, edema peripheral, insomnia, and vomiting was higher in the Dara‐Rd arm than in the Isa‐Kd arm; observed differences were not significant (Table 3). Post‐matching, rates of grade 3 or 4 diarrhea (0.6% vs. 7.1%; *p* = 0.0002) and febrile neutropenia (0.3% vs. 6.0%; *p* = 0.0045) were significantly lower in the Isa‐Kd than in the Dara‐Rd arm. Conversely, a higher rate of grade 3 or 4 pneumonia was observed in the Isa‐Kd arm than in the Dara‐Rd arm, whereas the proportion of grade 3 or 4 fatigue was higher in the Dara‐Rd arm than in the Isa‐Kd arm, though these differences were not significant (Table 3).

**TABLE 3:** Matching‐adjusted indirect comparison results (after matching) for Isa‐Kd versus Dara‐Rd in patients with relapsed multiple myeloma: All‐grade and grade 3 or 4 TEAEs.

	Isa‐Kd	Dara‐Rd	Odds ratio	*p*‐value
	TEAE rate (95% CI)	TEAE rate (95% CI)	(95% CI)	
Any TEAE leading to treatment discontinuation	9.0% (3.9%–19.4%)	12.0% (8.5%–16.4%)	0.73 (0.30–1.73)	0.4712
All‐grade TEAEs				
Asthenia	21.1% (12.5%–33.5%)	18.0% (13.7%–23.0%)	1.22 (0.69–2.17)	0.4999
Back pain	20.2% (11.9%–32.3%)	20.5% (15.9%–25.7%)	0.98 (0.56–1.74)	0.9561
Bronchitis	19.1% (11.5%–29.8%)	18.7% (14.4%–23.8%)	1.02 (0.59–1.77)	0.9378
Constipation	2.1% (0.8%–5.4%)	31.1% (25.7%–36.8%)	0.05 (0.02–0.13)	<0.0001*
Cough	17.0% (9.0%–29.9%)	32.2% (26.7%–37.9%)	0.43 (0.23–0.83)	0.0110*
Diarrhea	32.6% (22.4%–44.7%)	50.9% (44.9%–56.8%)	0.47 (0.31–0.71)	0.0004*
Dyspnea	27.7% (17.8%–40.3%)	20.9% (16.3%–26.1%)	1.45 (0.88–2.39)	0.1424
Edema peripheral	13.5% (7.0%–24.4%)	18.7% (14.4%–23.8%)	0.68 (0.34–1.34)	0.2631
Fatigue	23.7% (15.5%–34.5%)	36.4% (30.8%–42.3%)	0.54 (0.34–0.86)	0.0087*
Febrile neutropenia	0.3% (0.0%–2.4%)	6.0% (3.5%–9.4%)	0.05 (0.01–0.40)	0.0045*
Headache	17.9% (10.0%–30.0%)	15.2% (11.2%–19.9%)	1.22 (0.65–2.30)	0.5387
Insomnia	17.1% (10.1%–27.5%)	23.7% (18.8%–29.1%)	0.66 (0.38–1.16)	0.1522
Muscle spasms	13.4% (6.4%–25.8%)	28.6% (23.4%–34.3%)	0.38 (0.18–0.81)	0.0113*
Nasopharyngitis	14.0% (7.7%–24.2%)	29.7% (24.4%–35.4%)	0.39 (0.21–0.72)	0.0025*
Nausea	12.7% (6.9%–22.3%)	26.9% (21.8%–32.4%)	0.40 (0.21–0.75)	0.0043*
Pneumonia	22.3% (14.3%–33.0%)	20.5% (15.9%–25.7%)	1.11 (0.68–1.83)	0.6725
Pyrexia	7.3% (3.0%–17.0%)	23.7% (18.8%–29.1%)	0.26 (0.10–0.64)	0.0034*
Upper respiratory tract infection	41.8% (30.5%–54.1%)	37.1% (31.5%–43.0%)	1.22 (0.82–1.81)	0.3306
Vomiting	11.9% (5.9%–22.6%)	18.4% (14.0%–23.4%)	0.60 (0.29–1.25)	0.1726
Grade 3 or 4 TEAEs				
Diarrhea	0.6% (0.2%–2.2%)	7.1% (4.4%–10.7%)	0.08 (0.02–0.31)	0.0002*
Fatigue	3.0% (0.9%–9.4%)	6.4% (3.8%–9.9%)	0.46 (0.13–1.59)	0.2199
Febrile neutropenia	0.3% (0.0%–2.4%)	6.0% (3.5%–9.4%)	0.05 (0.01–0.40)	0.0045*
Pneumonia	16.1% (9.2%–26.6%)	12.0% (8.5%–16.4%)	1.40 (0.75–2.64)	0.2921

Abbreviations: CI, confidence interval; Dara‐Rd, daratumumab + lenalidomide + dexamethasone; Isa‐Kd, isatuximab + carfilzomib + dexamethasone; TEAE, treatment‐emergent adverse event.

* Statistically significant at a 0.05 level.

## 4 DISCUSSION

In the current clinical practice, lenalidomide‐based regimens, such as Dara‐Rd, are considered the go‐to regimen in the 2 L+ setting for patients with RRMM who experienced the first relapse and are frequently used in non‐lenalidomide‐refractory patients until progression.^22^, ^23^, ^24^ Immunotherapy drugs are being broadly used in the treatment of RRMM. Daratumumab was the first anti‐CD38 mAb to receive the FDA approval for patients with MM,^43^ later joined by another anti‐CD38 mAb, isatuximab, approved for use in patients with RRMM.^12^ Given the absence of published evidence from head‐to‐head clinical trials, we conducted the current MAIC analysis in patients with RRMM to understand the comparative efficacy and safety of Isa‐Kd versus Dara‐Rd. This MAIC analysis suggests that Isa‐Kd may represent an important new treatment option offering improved efficacy outcomes with a tolerable safety profile for non‐lenalidomide refractory patients at early relapse compared with Dara‐Rd, a lenalidomide‐based regimen.

Matching‐adjusted indirect comparison can adjust for differences between trial populations that are potential baseline treatment effect modifiers and prognostic factors that might otherwise bias the comparison.^32^, ^44^ After matching adjustment, all baseline characteristics were well balanced between the Isa‐Kd and Dara‐Rd populations except for patients with more than three prior LoT (0.0% vs. 4.9%) as it was not possible to match this factor given that the IKEMA trial did not include patients with more than three prior LoT. However, this difference is not likely to significantly impact the MAIC results, given the small number of these patients.

In this MAIC analysis, PFS was significantly better with Isa‐Kd than Dara‐Rd (pre‐ and post‐match HR: 0.66; *p* = 0.1002 vs. 0.46; *p* = 0.0155) after adjusting for differences in the inclusion criteria and baseline characteristics. The matching adjustment increased the estimated benefit of Isa‐Kd versus Dara‐Rd for OS (pre‐ and post‐match HR: 0.60; *p* = 0.1721 vs. 0.47; *p* = 0.0798), although it was statistically non‐significant. The existing literature suggests that an improved response (e.g., VGPR rate) is an important indicator of prognosis in patients with MM and is correlated with improvements in other efficacy outcomes, such as PFS and OS.^45^ A numerical improvement in VGPR or better rates was observed with Isa‐Kd compared with Dara‐Rd, although it was statistically non‐significant (pre‐ and post‐match OR: 1.00; *p* = 0.9837 vs. 1.53; *p* = 0.1252). The comparative efficacy results from this MAIC analysis should be interpreted with the caveat as the results from both clinical trials are immature. It will be important to update the recent analyses as extended follow‐up data become available to understand the long‐term survival estimates better.

In terms of safety profile, Isa‐Kd was well tolerated and demonstrated significantly lower rates of several all‐grade (0.3% vs. 6.0%; *p* = 0.0045) and grade 3 or 4 (0.3% vs. 6.0%; *p* = 0.0045) TEAEs than Dara‐Rd. Although higher rates were observed for a few TEAEs with Isa‐Kd than Dara‐Rd, the differences were statistically non‐significant. Febrile neutropenia is a recognized complication in cancer treatment that could result in hospitalization and could substantially impact treatment outcomes due to delays in a treatment cycle, dose reductions, or treatment discontinuations.^46^ A retrospective chart review study among adult patients with cancer and diagnosis of febrile neutropenia between 2010 and 2013 reported a hospital mortality rate of 17.3% and the 30‐day mortality rate of 20.5%, suggesting that febrile neutropenia in hospitalized patients could result in a significant mortality rate.^47^ In combination with dexamethasone, lenalidomide is one of the most frequently used treatment options in patients with MM though this combination has reported one of the highest incidences of neutropenia.^48^ Additionally, neutropenia has been predominantly reported for Dara‐Rd.^48^ In the POLLUX trial, a higher proportion of patients in the daratumumab group (51.9%) reported neutropenia than those in the control group, that is, Rd (37.0%).^18^ Though the rationale for this mechanism remains unclear, the target of daratumumab, CD38 (target epitope is different than that of isatuximab), is known to regulate neutrophil chemotaxis and is present on myeloid stem cells.^48^, ^49^, ^50^

Both isatuximab and daratumumab are anti‐CD38 mAb; however, isatuximab differentiates itself from daratumumab in terms of key mechanistic differences. At present, isatuximab is the only anti‐CD38 mAb that could induce direct apoptosis in MM cells, although only in patients with elevated CD38 expression, whereas daratumumab may induce apoptosis, but only in the presence of cross‐linking agents.^13^, ^51^, ^52^, ^53^, ^54^, ^55^ Isatuximab was shown to inhibit CD38 enzymatic activity more effectively than daratumumab.^56^, ^57^ In terms of the dosing schedule, isatuximab is administered every other week starting from cycle 2, whereas daratumumab is started from cycle 3.^14^, ^18^, ^36^ After six cycles, isatuximab is continued to be administered every other week, whereas daratumumab is administered once a month.^12^, ^17^ Additionally, the difference between carfilzomib and lenalidomide might have contributed to better efficacy outcomes with Isa‐Kd than with Dara‐Rd.^58^ Some of these possibilities, including the mechanism of action, dosing schedule of isatuximab, and enhanced antitumor effects of combination therapies, might have contributed to the augmented efficacy of Isa‐Kd compared with Dara‐Rd. However, further comparative evidence is warranted to draw definite conclusions.

The association of mAbs targeting CD38 with carfilzomib has been well studied. Two randomized phase 3 trials, that is, IKEMA (Kd vs. Kd with isatuximab) and CANDOR (Kd vs. Kd with daratumumab), are quite similar with a CD38‐directed mAb paired with the Kd backbone (control arm) in patients with RRMM with a median of two prior LoT.^14^, ^59^ Both trials have a similar design, and the proportion of lenalidomide‐refractory patients (32%–33%) was also comparable.^14^, ^59^ Although cross‐trial comparisons of other anti‐CD38 PI triplet combinations in this patient population should be interpreted with caution, the HR for PFS in the isatuximab group of IKEMA (median follow‐up 20.7 months: HR [95% CI]: 0.53 [0.32–0.89]) was numerically more favorable than in the CANDOR study (median follow‐up 16.9 months, HR [95% CI]: 0.63 [0.46–0.85]; median follow‐up 28.6 months, HR [95% CI]: 0.59 [0.45–0.78]).^14^, ^59^, ^60^ However, definite conclusions can only be drawn when longer follow‐up data will be available for both the IKEMA and CANDOR trials.

There are several limitations to this MAIC analysis that should be noted while considering its results, many of which are common to all MAICs. Given the absence of IPD for the POLLUX trial, it is challenging to quantify the amount of residual bias in the treatment effect estimates, and it might be possible that some confounding variables may have remained unbalanced. Another drawback of the current study is the limited follow‐up data from both clinical studies (median follow‐up: IKEMA [20.7 months]; POLLUX [44.3 months for PFS, 13.5 months for OS, and 25.4 months for VGPR or better]); therefore, uncertainties may exist in the current analysis based on the available data. After matching adjustment, there was a substantial reduction in ESS from the original sample size (around 60%, from 112 to 66.2). This reduction reflects that the Isa‐Kd population in IKEMA was among other differences with higher cytogenic risk (24% vs. 12% before matching) and more refractory to previous line (35% vs. 28% before matching) than the Dara‐Rd treated population in POLLUX, and thus, moderate overlapping with the POLLUX patient population included in this MAIC. In addition, this is unsure whether real‐world patients present distributions closest to the POLLUX or IKEMA patient population profiles, and whether this could influence the results as we do not have the POLLUX individual patient data to explore what would be the results with a patient profile closest to IKEMA.

In conclusion, this MAIC demonstrated a significant benefit in PFS, and a statistically non‐significant trend was observed for OS and VGPR or better rates in favor of Isa‐Kd compared with Dara‐Rd. Furthermore, Isa‐Kd demonstrated an acceptable safety profile with significantly lower rates of several TEAEs, including febrile neutropenia, compared with Dara‐Rd. These results support the value of Isa‐Kd as an efficacious treatment option in an early relapse in non‐lenalidomide refractory patients compared with Dara‐Rd. An updated analysis using extended follow‐up data is further warranted to draw definitive conclusions.

## AUTHOR CONTRIBUTIONS

**Joshua Richter:** Conceptualization (equal); investigation (equal); methodology (equal); writing – original draft (equal); writing – review and editing (equal). **Peggy L. Lin:** Conceptualization (equal); funding acquisition (equal); investigation (equal); project administration (equal); supervision (equal); validation (equal); writing – original draft (equal); writing – review and editing (equal). **Viviana Garcia‐Horton:** Data curation (equal); formal analysis (equal); investigation (equal); resources (equal); software (equal); writing – original draft (equal); writing – review and editing (equal). **Patricia Guyot:** Conceptualization (equal); investigation (equal); project administration (equal); resources (equal); writing – original draft (equal); writing – review and editing (equal). **Erin Singh:** Conceptualization (equal); funding acquisition (equal); project administration (equal); resources (equal); supervision (equal); visualization (equal); writing – original draft (equal); writing – review and editing (equal). **Zheng‐Yi Zhou:** Conceptualization (equal); data curation (equal); formal analysis (equal); investigation (equal); methodology (equal); project administration (equal); software (equal); writing – original draft (equal); writing – review and editing (equal). **Mark Sievert:** Conceptualization (equal); funding acquisition (equal); investigation (equal); project administration (equal); resources (equal); writing – original draft (equal); writing – review and editing (equal). **Riley Taiji:** Conceptualization (equal); data curation (equal); formal analysis (equal); investigation (equal); methodology (equal); software (equal); validation (equal); writing – original draft (equal); writing – review and editing (equal).

## FUNDING INFORMATION

This study was funded by Sanofi.

## CONFLICT OF INTEREST STATEMENT

Joshua Richter reported receiving speaker's bureau fee from Janssen, Celgene, and Adaptive Biotechnologies and is a member of consulting/advisory board of Celgene, Janssen, BMS, Karyopharm, Antengene, Sanofi, X4 Pharmaceuticals, Oncopeptides, Adaptive Biotechnologies, Secura Bio, and Astra Zeneca. Peggy L. Lin, Patricia Guyot, Erin Singh, and Mark Sievert reported being employed by and holding stock in Sanofi. Viviana Garcia‐Horton and Zheng‐Yi Zhou reported being employed by Analysis Group, which received funding from Sanofi for this research. Riley Taiji was a former employee of Analysis Group, which received funding from Sanofi for this research.

## ETHICS APPROVAL STATEMENT

Not applicable. The Declaration of Helsinki and its amendments do not apply to this manuscript, as data used for analyses were completely de‐identified (for isatuximab in combination with carfilzomib and dexamethasone [IsaKd]) or abstracted from relevant publications (for daratumumab, in combination with carfilzomib and dexamethasone [DaraRd]). The current study assesses comparative efficacy through statistical analyses and does not involve any clinical intervention. The randomized controlled trials (IKEMA and POLLUX trials), which were the sources of the data used for statistical analyses in the current study, were conducted according to the principles of the Declaration of Helsinki, and its study protocol was approved by ethics committee/IRB for each center with informed consent from participants.

## PATIENT CONSENT STATEMENT

Not applicable. The written informed consent is not applicable as this study is a matching‐adjusted indirect comparison analysis of individual patient‐level data for isatuximab in combination with carfilzomib and dexamethasone obtained from the IKEMA trial (NCT03275285) versus published aggregate data for daratumumab, in combination with carfilzomib and dexamethasone obtained from the POLLUX trial publications.

## PERMISSION TO REPRODUCE MATERIAL FROM OTHER SOURCES

Not applicable.

## CLINICAL TRIAL REGISTRATION

NCT03275285.

## PRIOR PRESENTATION

Parts of the data were presented as posters at 18th International Myeloma Workshop, September 8–11, 2021 (Abstract: 1073241). Detailed citation: Richter J, et al. P‐213: A matching‐adjusted indirect comparison of isatuximab plus carfilzomib and dexamethasone versus daratumumab plus lenalidomide and dexamethasone for relapsed multiple myeloma. Clin Lymphoma Myeloma Leuk. 2021;21(Suppl 2):S156–S157. https://doi.org/10.1016/S2152‐2650(21)02340‐5. Congress URL: https://www.myelomasociety.org/events/imw‐vienna/. 63rd ASH Annual Meeting and Exposition, December 11–14, 2021 (Publication Number: 1962). Detailed citation: Richter J, et al. 1962 A matching‐adjusted indirect comparison of isatuximab plus carfilzomib and dexamethasone versus daratumumab plus lenalidomide and dexamethasone for relapsed multiple myeloma. 63rd ASH Annual Meeting and Exposition, December 11–14, 2021; Available from: https://ash.confex.com/ash/2021/webprogram/Paper150299.html. Congress URL: https://www.hematology.org/meetings/annual‐meeting.

## Data Availability

The datasets used and/or analyzed during the current study are available from the corresponding author on reasonable request.

## References

[1] van de Donk NWCJ , Pawlyn C , Yong KL . Multiple myeloma. Lancet. 2021;397(10272):410‐427. 33516340 10.1016/S0140-6736(21)00135-5

[2] Fairfield H , Falank C , Avery L , Reagan MR . Multiple myeloma in the marrow: pathogenesis and treatments. Ann NY Acad Sci. 2016;1364(1):32‐51. 27002787 10.1111/nyas.13038 PMC4806534

[3] Kazandjian D . Multiple myeloma epidemiology and survival: a unique malignancy. Semin Oncol. 2016;43(6):676‐681. 28061985 10.1053/j.seminoncol.2016.11.004 PMC5283695

[4] Globocan . Multiple myeloma fact sheet; 2021. Accessed May 18, 2022. https://gco.iarc.fr/today/data/factsheets/cancers/35‐Multiple‐myeloma‐fact‐sheet.pdf

[5] Surveillance Epidemiology and End Results Program . Cancer Stat Facts: Myeloma; 2021. Accessed May 18, 2022. https://seer.cancer.gov/statfacts/html/mulmy.html

[6] Cancer Research UK . Myeloma Statistics; 2021. Accessed May 18, 2022. https://www.cancerresearchuk.org/health‐professional/cancer‐statistics/statistics‐by‐cancer‐type/myeloma#heading‐Zero

[7] Cancer Research UK . Myeloma incidence trends over time; 2021. Accessed May 18, 2022. https://www.cancerresearchuk.org/health‐professional/cancer‐statistics/statistics‐by‐cancer‐type/myeloma/incidence#heading‐Two

[8] Kumar SK , Dispenzieri A , Lacy MQ , et al. Continued improvement in survival in multiple myeloma: changes in early mortality and outcomes in older patients. Leukemia. 2014;28(5):1122‐1128. 24157580 10.1038/leu.2013.313 PMC4000285

[9] Lee JH , Kim S‐H . Treatment of relapsed and refractory multiple myeloma. Blood Res. 2020;55(S1):S43‐S53. 32719176 10.5045/br.2020.S008 PMC7386890

[10] Nishida H , Yamada T . Monoclonal antibody therapies in multiple myeloma: a challenge to develop novel targets. J Oncol. 2019;2019:6084012‐6084010. 31781214 10.1155/2019/6084012 PMC6875016

[11] Saltarella I , Desantis V , Melaccio A , et al. Mechanisms of resistance to anti‐CD38 daratumumab in multiple myeloma. Cell. 2020;9(1):167. 10.3390/cells9010167 PMC7017193 31936617

[12] U.S. Food and Drug Administration . Highlights of Prescribing information: SARCLISA® [Reference ID: 4771454]; 2021. Accessed May 18, 2022. https://www.accessdata.fda.gov/drugsatfda_docs/label/2021/761113s003lbl.pdf

[13] Deckert J , Wetzel M‐C , Bartle LM , et al. SAR650984, a novel humanized CD38‐targeting antibody, demonstrates potent antitumor activity in models of multiple myeloma and other CD38+ hematologic malignancies. Clin Cancer Res. 2014;20(17):4574‐4583. 24987056 10.1158/1078-0432.CCR-14-0695

[14] Moreau P , Dimopoulos M‐A , Mikhael J , et al. Isatuximab, carfilzomib, and dexamethasone in relapsed multiple myeloma (IKEMA): a multicentre, open‐label, randomised phase 3 trial. Lancet. 2021;397(10292):2361‐2371. 34097854 10.1016/S0140-6736(21)00592-4

[15] European Medicines Agency . Sarclisa product information; 2021. Accessed May 18, 2022. https://www.ema.europa.eu/en/documents/product‐information/sarclisa‐epar‐product‐information_en.pdf

[16] de Weers M , Tai Y‐T , van der Veer MS , et al. Daratumumab, a novel therapeutic human CD38 monoclonal antibody, induces killing of multiple myeloma and other hematological tumors. J Immunol. 2011;186(3):1840‐1848. 21187443 10.4049/jimmunol.1003032

[17] U.S. Food and Drug Administration . Highlights of Prescribing Information: DARZALEX®; 2021. Accessed May 18, 2022. https://www.janssenlabels.com/package‐insert/product‐monograph/prescribing‐information/DARZALEX‐pi.pdf

[18] Dimopoulos MA , Oriol A , Nahi H , et al. Daratumumab, lenalidomide, and dexamethasone for multiple myeloma. N Engl J Med. 2016;375(14):1319‐1331. 27705267 10.1056/NEJMoa1607751

[19] European Medicines Agency . Darzalex, Summary of Product Characteristics; 2021. Accessed May 18, 2022. https://www.ema.europa.eu/en/documents/product‐information/darzalex‐epar‐product‐information_en.pdf

[20] U.S. Food and Drug Administration . FDA approves carfilzomib and daratumumab with dexamethasone for multiple myeloma; 2020. Accessed May 18, 2022. https://www.fda.gov/drugs/resources‐information‐approved‐drugs/fda‐approves‐carfilzomib‐and‐daratumumab‐dexamethasone‐multiple‐myeloma

[21] Sonneveld P , Broijl A . Treatment of relapsed and refractory multiple myeloma. Haematologica. 2016;101(4):396‐406. 27033237 10.3324/haematol.2015.129189 PMC5004403

[22] Gershon A , Liu Y , James S , Williamson M , Hong W‐J , Sarsour K . Real world data (RWD) treatment patterns and sequencing of patients with multiple myeloma (MM). Blood. 2020;136(Suppl 1):46.

[23] Nathwani N , Bertamini L , Banerjee R , Gay F , Shah N , Krishnan A . When and how to treat relapsed multiple myeloma. Am Soc Clin Oncol Educ Book. 2021;41:358‐375. 34010043 10.1200/EDBK_320129

[24] Rajkumar SV , Kumar S . Multiple myeloma current treatment algorithms. Blood Cancer J. 2020;10(9):94. 32989217 10.1038/s41408-020-00359-2 PMC7523011

[25] Legarda MA , Cejalvo MJ , de la Rubia J . Recent advances in the treatment of patients with multiple myeloma. Cancer. 2020;12(12):3576. 10.3390/cancers12123576 PMC7761116 33265952

[26] Bird SA , Boyd K . Multiple myeloma: an overview of management. Palliat Care Soc Pract. 2019;13:1178224219868235. 32215370 10.1177/1178224219868235 PMC7065505

[27] Ninkovic S , Quach H . Shaping the treatment paradigm based on the current understanding of the pathobiology of multiple myeloma: an overview. Cancer. 2020;12(11):3488. 10.3390/cancers12113488 PMC7700434 33238653

[28] Majithia N , Rajkumar SV , Lacy MQ , et al. Early relapse following initial therapy for multiple myeloma predicts poor outcomes in the era of novel agents. Leukemia. 2016;30(11):2208‐2213. 27211270 10.1038/leu.2016.147 PMC5541860

[29] Kumar SK , Dimopoulos MA , Kastritis E , et al. Natural history of relapsed myeloma, refractory to immunomodulatory drugs and proteasome inhibitors: a multicenter IMWG study. Leukemia. 2017;31(11):2443‐2448. 28620163 10.1038/leu.2017.138

[30] Usmani S , Ahmadi T , Ng Y , et al. Analysis of real‐world data on overall survival in multiple myeloma patients with ≥3 prior lines of therapy including a proteasome inhibitor (PI) and an immunomodulatory drug (IMiD), or double refractory to a PI and an IMiD. Oncologist. 2016;21(11):1355‐1361. 27486203 10.1634/theoncologist.2016-0104 PMC5189616

[31] Phillippo DM , Ades A , Dias S , Palmer S , Abrams KR , Welton NJ . NICE DSU technical support document 18: methods for population‐adjusted indirect comparisons in submissions to NICE. Report by the Decision Support Unit. Vol. 18. Decision Support Unit, ScHARR, University of Sheffield; 2016:81.

[32] Phillippo DM , Ades AE , Dias S , Palmer S , Abrams KR , Welton NJ . Methods for population‐adjusted indirect comparisons in health technology appraisal. Med Decis Making. 2018;38(2):200‐211. 28823204 10.1177/0272989X17725740 PMC5774635

[33] Jiang Y , Ni W . Performance of unanchored matching‐adjusted indirect comparison (MAIC) for the evidence synthesis of single‐arm trials with time‐to‐event outcomes. BMC Med Res Methodol. 2020;20(1):241. 32993519 10.1186/s12874-020-01124-6 PMC7526361

[34] Rodriguez‐Otero P , Ayers D , Cope S , et al. Matching adjusted indirect comparisons of efficacy outcomes for idecabtagene vicleucel (ide‐cel, bb2121) versus selinexor + dexamethasone and belantamab mafodotin in relapsed and refractory multiple myeloma. Leuk Lymph. 2021;62(10):2482‐2491. 10.1080/10428194.2021.1913143 33896344

[35] Martin T , Usmani SZ , Schecter JM , et al. Matching‐adjusted indirect comparison of efficacy outcomes for ciltacabtagene autoleucel in CARTITUDE‐1 versus idecabtagene vicleucel in KarMMa for the treatment of patients with relapsed or refractory multiple myeloma. Curr Med Res Opin. 2021;37(10):1779‐1788. 34256668 10.1080/03007995.2021.1953456

[36] Moreau P , Dimopoulos MA , Yong K , et al. Isatuximab plus carfilzomib/dexamethasone versus carfilzomib/dexamethasone in patients with relapsed/refractory multiple myeloma: IKEMA phase III study design. Future Oncol. 2020;16(2):4347‐4358. 31833394 10.2217/fon-2019-0431

[37] Dimopoulos MA , San‐Miguel J , Belch A , et al. Daratumumab plus lenalidomide and dexamethasone versus lenalidomide and dexamethasone in relapsed or refractory multiple myeloma: updated analysis of POLLUX. Haematologica. 2018;103(12):2088‐2096. 30237262 10.3324/haematol.2018.194282 PMC6269302

[38] IKEMA trial clinical study report [data on file].

[39] Bahlis NJ , Dimopoulos MA , White DJ , et al. Daratumumab plus lenalidomide and dexamethasone in relapsed/refractory multiple myeloma: extended follow‐up of POLLUX, a randomized, open‐label, phase 3 study. Leukemia. 2020;34(7):1875‐1884. 32001798 10.1038/s41375-020-0711-6 PMC7326710

[40] Mark Mitchell BMaTWea . Engauge Digitizer Software. Accessed May 18, 2022. http://markummitchell.github.io/engauge‐digitizer

[41] Guyot P , Ades AE , Ouwens MJNM , Welton NJ . Enhanced secondary analysis of survival data: reconstructing the data from published Kaplan‐Meier survival curves. BMC Med Res Methodol. 2012;12(1):9. 22297116 10.1186/1471-2288-12-9 PMC3313891

[42] Phillippo DM , Dias S , Elsada A , Ades AE , Welton NJ . Population adjustment methods for indirect comparisons: a review of National Institute for Health And Care Excellence Technology appraisals. Int J Technol Assess Health Care. 2019;35(3):221‐228. 31190671 10.1017/S0266462319000333 PMC6650293

[43] D'Agostino M , Innorcia S , Boccadoro M , Bringhen S . Monoclonal antibodies to treat multiple myeloma: a dream come true. Int J Mol Sci. 2020;21(21):8192. 33139668 10.3390/ijms21218192 PMC7662679

[44] Signorovitch JE , Sikirica V , Erder MH , et al. Matching‐adjusted indirect comparisons: a new tool for timely comparative effectiveness research. Value Health. 2012;15(6):940‐947. 22999145 10.1016/j.jval.2012.05.004

[45] Dubey A , Khatri S , Maggo S , Singh N , Sharma D . Daratumumab plus carfilzomib: an optimistic approach in relapsed/refractory multiple myeloma. Indian J Med Paediatr Oncol. 2020;41:846‐849.

[46] Kuderer NM , Dale DC , Crawford J , Cosler LE , Lyman GH . Mortality, morbidity, and cost associated with febrile neutropenia in adult cancer patients. Cancer. 2006;106(10):2258‐2266. 16575919 10.1002/cncr.21847

[47] Hosiriluck N , Klomjit S , Rassameehiran S , Sutamtewagul G , Tijani L , Radhi S . Prognostic factors for mortality with febrile neutropenia in hospitalized patients. Southwest Respir Crit Care Chronicles. 2015;3:3.

[48] Leleu X , Gay F , Flament A , Allcott K , Delforge M . Incidence of neutropenia and use of granulocyte colony‐stimulating factors in multiple myeloma: is current clinical practice adequate? Ann Hematol. 2018;97(3):387‐400. 29282494 10.1007/s00277-017-3191-7 PMC5797221

[49] Partida‐Sánchez S , Cockayne DA , Monard S , et al. Cyclic ADP‐ribose production by CD38 regulates intracellular calcium release, extracellular calcium influx and chemotaxis in neutrophils and is required for bacterial clearance in vivo. Nat Med. 2001;7(11):1209‐1216. 11689885 10.1038/nm1101-1209

[50] Faramarz N . Rao PN. Acute myeloid Leukemia. In: Naeim F , Rao PN , Grody WW , eds. Hematopathology Morphology, Immunophenotype, Cytogenetics, and Molecular Approaches. 1st ed. UK Academic Press ‐ Elsevier; 2008:207‐255.

[51] van de Donk N , Usmani SZ . CD38 antibodies in multiple myeloma: mechanisms of action and modes of resistance. Front Immunol. 2018;9:2134. 30294326 10.3389/fimmu.2018.02134 PMC6158369

[52] van de Donk NW , Janmaat ML , Mutis T , et al. Monoclonal antibodies targeting CD 38 in hematological malignancies and beyond. Immunol Rev. 2016;270(1):95‐112. 26864107 10.1111/imr.12389 PMC4755228

[53] Jiang H , Acharya C , An G , et al. SAR650984 directly induces multiple myeloma cell death via lysosomal‐associated and apoptotic pathways, which is further enhanced by pomalidomide. Leukemia. 2016;30(2):399‐408. 26338273 10.1038/leu.2015.240

[54] Martin TG , Corzo K , Chiron M , et al. Therapeutic opportunities with pharmacological inhibition of CD38 with isatuximab. Cell. 2019;8(12):1522. 10.3390/cells8121522 PMC6953105 31779273

[55] Moreno L , Perez C , Zabaleta A , et al. The mechanism of action of the anti‐cd38 monoclonal antibody isatuximab in multiple myeloma. Clin Cancer Res. 2019;25(10):3176‐3187. 30692097 10.1158/1078-0432.CCR-18-1597

[56] van Bueren JL , Jakobs D , Kaldenhoven N , et al. Direct in vitro comparison of daratumumab with surrogate analogs of CD38 antibodies MOR03087, SAR650984 and Ab79. Blood. 2014;124(21):3474.

[57] Mikhael J , Belhadj‐Merzoug K , Hulin C , et al. A phase 2 study of isatuximab monotherapy in patients with multiple myeloma who are refractory to daratumumab. Blood Cancer J. 2021;11(5):89. 33980831 10.1038/s41408-021-00478-4 PMC8116334

[58] Rifkin RM , Medhekar R , Amirian ES , et al. A real‐world comparative analysis of carfilzomib and other systemic multiple myeloma chemotherapies in a US community oncology setting. Ther Adv Hematol. 2019;10:2040620718816699. 30719266 10.1177/2040620718816699 PMC6348507

[59] Dimopoulos M , Quach H , Mateos MV , et al. Carfilzomib, dexamethasone, and daratumumab versus carfilzomib and dexamethasone for patients with relapsed or refractory multiple myeloma (CANDOR): results from a randomised, multicentre, open‐label, phase 3 study. Lancet. 2020;396(10245):186‐197. 32682484 10.1016/S0140-6736(20)30734-0

[60] Dimopoulos M , Quach H , Mateos MV , Landgren O , Leleu X , Siegel D , et al. 2325 carfilzomib, dexamethasone, and daratumumab versus carfilzomib and dexamethasone in relapsed or refractory multiple myeloma: updated efficacy and safety results of the phase 3 candor study. 62nd ASH Annual Meeting and Exposition; 2020.

